# Complete Genome Sequence of *Salmonella enterica* subsp. *enterica* Serovar Indiana C629, a Carbapenem-Resistant Bacterium Isolated from Chicken Carcass in China

**DOI:** 10.1128/genomeA.00662-16

**Published:** 2016-07-14

**Authors:** Wei Wang, Feng Liu, Zixin Peng, Fengqin Li, Aiguo Ma

**Affiliations:** a Medical College, Qingdao University, Qingdao, People’s Republic of China; b Microbiology Laboratory, China National Centre for Food Safety Risk Assessment, Chaoyang District, Beijing, People’s Republic of China; c Pharmaceutical Department, Qingdao Hiser Medical Center, Qingdao, People’s Republic of China

## Abstract

The carbapenem-resistant *Salmonella enterica* subsp. *enterica* serovar Indiana strain C629 was isolated from a chicken carcass collected from a slaughterhouse in Qingdao, China. The complete genome sequence of C629 contains a circular 4,791,723-bp chromosome and a circular 210,106-bp plasmid. Genes involved in carbapenem resistance of this bacterium were identified by whole-genome analysis.

## GENOME ANNOUNCEMENT

*Salmonella enterica* is a major global foodborne pathogen, causing life-threatening infections (1). Carbapenems, including imipenem and meropenem, have been considered for the treatment of *S. enterica* infections because they are not hydrolyzed by most of the serine β-lactamases (2). For several years, the resistance of *S. enterica* to expanded-spectrum carbapenems has increasingly been reported (3, 4). NDM-1 has been reported in two strains of *Salmonella* spp., which were isolated from feces and urine specimens during screening for multidrug-resistant bacteria in patients from India (5, 6). In this study, carbapenem-resistant *Salmonella enterica* subsp. *enterica* serovar Indiana C629 was isolated from a chicken carcass collected from a slaughterhouse in Qingdao, China. The complete genome sequence of the C629 strain was determined in order to provide the genetic basis for carbapenem resistance mechanisms of *S*. Indiana in the future.

Whole-genome sequencing of *S*. Indiana C629 was performed using the Pacific Biosciences RS II sequencing platform (Pacific Biosciences, Menlo Park, CA, USA). A 10-kb SMRTbell library was prepared from sheared genomic DNA using a 10-kb template library preparation workflow. Single-molecule real-time (SMRT) sequencing was conducted using the C4 sequencing chemistry and P6 polymerase with 1 SMRT cell. *De novo* assembly of the PacBio read sequences was carried out using continuous long reads (CLR), according to the Hierarchical Genome Assembly Process (HGAP) workflow (PacBioDevNet; Pacific Biosciences), as available in SMRT Analysis version 2.3. The complete genome sequence of *S*. Indiana C629 contains a circular 4,791,723-bp chromosome and a circular 210,106-bp plasmid (designated plasmid pRCW 1), with G+C contents of 52.08% and 48.56%, respectively. There are a total of 4,493 predicted genes in the chromosome, including 4,387 protein-coding genes, 22 tRNA-coding genes, and 84 rRNA-coding genes. There are 223 predicted protein-coding genes in plasmid pRCW 1.

The functions of the predicted proteins were annotated based on homologs compared against the NCBI-nr, Pfam, and KEGG databases. It was found, of all the proteins in *S*. Indiana C629, 3,623 proteins have homologues in the evolutionary genealogy of genes: Non-supervised Orthologous Groups (eggNOG) databases and assigned proper terms. The remaining proteins have no orthologous groups (e-value <1e - 5).

Genes related to carbapenem resistance have been annotated in the genome sequence. These genes or gene clusters will further explain their potential relevance in carbapenem resistance. Virulence genes and antibiotic genes are also predicted by Virulence Factor Database and Antibiotic Resistance Genes Database, respectively. In conclusion, the genome of *S*. Indiana C629 will enrich the carbapenem resistance genome database and facilitate the study of the carbapenem resistance mechanism.

### Nucleotide sequence accession numbers.

The complete genome sequence of *S*. Indiana C629 has been deposited at the GenBank under the accession numbers CP015724 (chromosome) and CP015725 (plasmid pRCW 1).
